# Supplementary figures and images for: Using Electrooculography and Electrodermal Activity During a Cold Pressor Test to Identify Physiological Biomarkers of State Anxiety: Feature-Based Algorithm Development and Validation Study
Source: JMIRx Med. 2025 Jul 10;6:e69472. doi: 10.2196/69472 (PMC12270033; doi:10.2196/69472)

Multimedia Appendix 1. Find Nearby Minimum Function


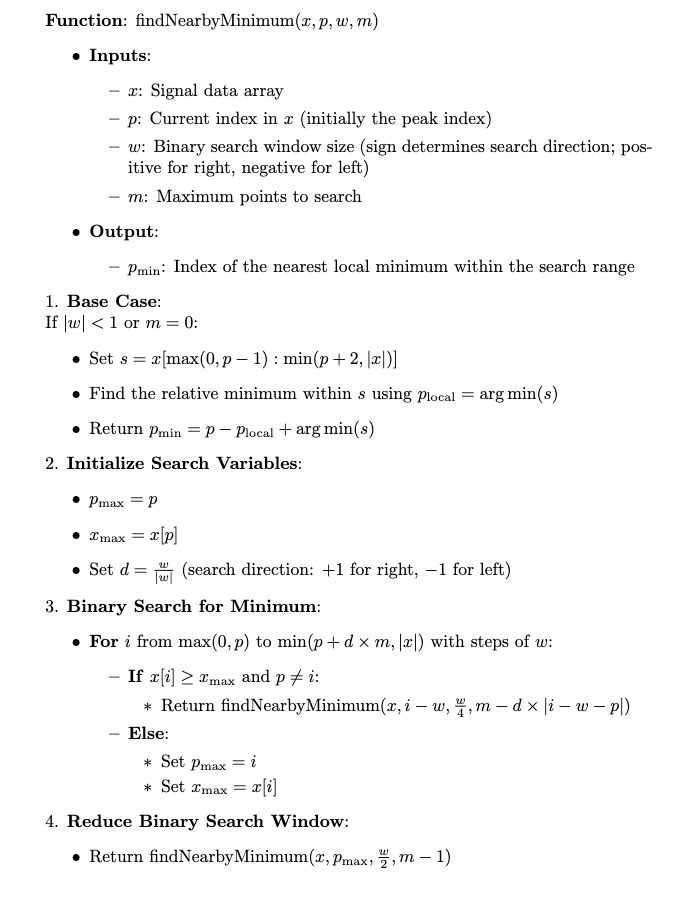

Supplement: Multimedia Appendix 1 [file xmed-v6-e69472-s001.docx]

Multimedia Appendix 5. Blink Duration Feature Culling


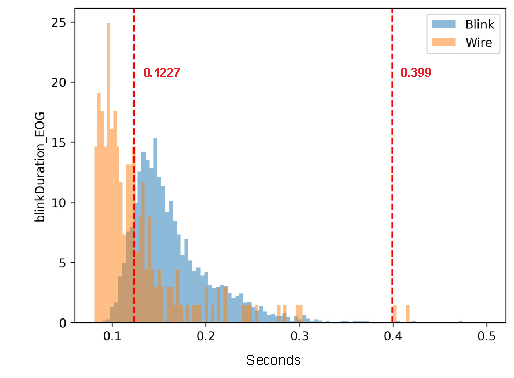

Supplement: Multimedia Appendix 5 [file xmed-v6-e69472-s005.docx]
