# Supplementary material for: Using Electrooculography and Electrodermal Activity During a Cold Pressor Test to Identify Physiological Biomarkers of State Anxiety: Feature-Based Algorithm Development and Validation Study
Source: JMIRx Med. 2025 Jul 10;6:e69472. doi: 10.2196/69472 (PMC12270033; doi:10.2196/69472)
Supplement: Multimedia Appendix 2 [file xmed-v6-e69472-s002.docx]

Multimedia Appendix 2. EMOCOLD Participant Survey Questionnaire

| **Survey Item** | **Scale** | **Dimension Evaluated** |
| --- | --- | --- |
| Upset | 1-5 | Negative Affectivity |
| Hostile | 1-5 | Negative Affectivity |
| Alert | 1-5 | Positive Affectivity |
| Ashamed | 1-5 | Negative Affectivity |
| Inspired | 1-5 | Positive Affectivity |
| Nervous | 1-5 | Negative Affectivity |
| Determined | 1-5 | Positive Affectivity |
| Attentive | 1-5 | Positive Affectivity |
| Active | 1-5 | Positive Affectivity |
| Afraid | 1-5 | Negative Affectivity |
| I feel calm | 1-4 | State Anxiety (Positive) |
| I feel secure | 1-4 | State Anxiety (Positive) |
| I am tense | 1-4 | State Anxiety (Negative) |
| I feel strained | 1-4 | State Anxiety (Negative) |
| I feel at ease | 1-4 | State Anxiety (Positive) |
| I feel upset | 1-4 | State Anxiety (Negative) |
| I am presently worrying over possible misfortunes | 1-4 | State Anxiety (Negative) |
| I feel satisfied | 1-4 | State Anxiety (Positive) |
| I feel frightened | 1-4 | State Anxiety (Negative) |
| I feel comfortable | 1-4 | State Anxiety (Positive) |
| I feel self-confident | 1-4 | State Anxiety (Positive) |
| I feel nervous | 1-4 | State Anxiety (Negative) |
| I am jittery | 1-4 | State Anxiety (Negative) |
| I feel indecisive | 1-4 | State Anxiety (Negative) |
| I am relaxed | 1-4 | State Anxiety (Positive) |
| I feel content | 1-4 | State Anxiety (Positive) |
| I am worried | 1-4 | State Anxiety (Negative) |
| I feel confused | 1-4 | State Anxiety (Negative) |
| I feel steady | 1-4 | State Anxiety (Positive) |

Scale Interpretations:

- 1-5 Scale (Items 1-10):
  - 1: Very slightly or not at all
  - 2: A little
  - 3: Moderately
  - 4: Quite a bit
  - 5: Extremely
- 1-4 Scale (Items 11-30):
  - 1: Not at all
  - 2: Somewhat
  - 3: Moderately so
  - 4: Very much so
