# Supplementary material for: Using Electrooculography and Electrodermal Activity During a Cold Pressor Test to Identify Physiological Biomarkers of State Anxiety: Feature-Based Algorithm Development and Validation Study
Source: JMIRx Med. 2025 Jul 10;6:e69472. doi: 10.2196/69472 (PMC12270033; doi:10.2196/69472)
Supplement: Multimedia Appendix 3 [file xmed-v6-e69472-s003.docx]

Multimedia Appendix 3. EDA Features Extracted

| **name** | **definition** |
| --- | --- |
| Signal Mean | The average voltage within a specific window of the electrodermal activity (EDA) signal. |
| Signal Standard Deviation | The standard deviation of the EDA signal within a window, indicating variability in the signal. |
| Signal Range | The range of values (difference between maximum and minimum) within the EDA signal window. |
| Velocity Mean | The average value of the first derivative of the EDA signal within the window, capturing the average rate of change. |
| Velocity Standard Deviation | The standard deviation of the first derivative, measuring the variability in the rate of change of the EDA signal. |
| Petrosian Fractal Dimension | The Petrosian fractal dimension of the EDA signal, used to quantify the complexity of the signal’s structure. |
| Higuchi Fractal Dimension | The Higuchi fractal dimension of the EDA signal, another measure of complexity, particularly suited for time series data. |
| DFA | Detrended Fluctuation Analysis of the EDA signal, which quantifies self-similarity and long-range correlations within the signal. |
| Katz Fractal Dimension | The Katz fractal dimension of the EDA signal, providing a measure of waveform complexity and irregularity. |
| Hjorth Activity (1^st^ Hjorth Parameter) | A measure of the signal’s power or variance, part of the Hjorth parameters used in time-domain analysis. |
| Hjorth Mobility (2^nd^ Hjorth Parameter) | The square root of the variance of the first derivative of the signal divided by the variance of the signal itself, measuring the signal’s frequency characteristics. |
| Hjorth Complexity (3^rd^ Hjorth Parameter) | A measure derived from Hjorth parameters, capturing the signal’s waveform complexity. |
| Variance of Rate of Change | The variance of the first derivative of the EDA signal, indicating variability in the signal’s rate of change. |
| Spectral Entropy | The entropy of the EDA signal’s power spectrum, representing the disorder or unpredictability within the frequency domain. |
| Permutation Entropy | The permutation entropy of the EDA signal, a nonlinear measure of signal complexity that is sensitive to dynamic changes in the signal’s structure. |
