# Supplementary material for: Using Electrooculography and Electrodermal Activity During a Cold Pressor Test to Identify Physiological Biomarkers of State Anxiety: Feature-Based Algorithm Development and Validation Study
Source: JMIRx Med. 2025 Jul 10;6:e69472. doi: 10.2196/69472 (PMC12270033; doi:10.2196/69472)
Supplement: Multimedia Appendix 4 [file xmed-v6-e69472-s004.docx]

Multimedia Appendix 4. EOG Features Extracted

| **name** | **definition** |
| --- | --- |
| Signal Height | The maximum amplitude of the EOG signal during an eye movement or blink peak. |
| X-Axis Deviation | Measures the horizontal difference between the x-coordinate of the signal's peak and the x-coordinate of the intersection of the upslope and downslope tangents, indicating asymmetry along the horizontal axis. |
| Y-Axis Deviation | Measures the vertical difference between the y-coordinate of the signal's peak and the y-coordinate of the intersection of the upslope and downslope tangents, reflecting differences in the steepness or curvature of the peak's slopes. |
| Symmetry Ratio | The ratio between tent deviations in X and Y axes, providing insight into blink symmetry or angle. |
| Closing Signal Range | The amplitude of the EOG signal during the full segment of the eye-closing phase. This is the range between the start of the peak rise and the point where the first derivative approaches zero after the closing segment. |
| Opening Signal Range | The amplitude of the EOG signal during the full segment of the eye-opening phase. This is the end of the closing segment to the baseline return, where the first derivative crosses zero after the peak. |
| Closing Duration | The interval between maximum velocity and amplitude during the blink, measuring blink speed. This is the second derivative peaks (indicating maximum velocity) before the first derivative returns to zero at the amplitude peak. |
| Closing Dynamics Ratio | The ratio of peak velocity to peak amplitude of the blink. This is the maximum velocity divided by Signal Height. |
| Blink Duration | The total duration of the blink event in the EOG signal. |
| Closing Tent Duration | The time taken to close the eye during the tenting phase of the blink. This is the time from the start of the upward slope until the first derivative peaks, indicating maximum closing speed. |
| Opening Tent Duration | This corresponds to the eye-opening duration during tenting. This is the time from the highest point of the blink (local maximum) to the return to baseline, indicated by a zero in the first derivative. |
| Closing Tent Duration by Proportion of Blink | The fraction of the blink duration taken by the eye-closing phase. This is the closing time tent duration divided by the total blink duration. |
| Opening Tent Duration by Proportion of Blink | The fraction of the blink duration taken by the eye-opening phase. This is the opening time tent duration divided by the total blink duration. |
| Blink Half-Close Duration | The time interval during which the eye is half-closed within the blink event. This is when the signal amplitude reaches half of Signal Height during both the closing and opening phases. |
| Blink Full-Close Duration | This corresponds to the duration for which the eyes are fully closed, or near the peak. This is the time from the end of the closing phase (peak of the signal) until the start of the opening phase, using where the first derivative approaches zero. |
| Full-Close Duration by Percentage of Blink | This corresponds to the percentage of the blink time during which the eyes are fully closed. Divide Blink Full-Close Duration by Blink Duration and multiply by 100. |
| Opening Acceleration to Peak Duration | This corresponds to a condensed duration measure of the blink, specifically calculated from the start of significant acceleration, the left acceleration maximum, to the end of the blink peak. |
| Velocity Recovery Duration | This metric corresponds to the duration of the blink segment where the signal returns to the same amplitude level as the initial velocity peak. This is the time interval from the left-side velocity peak to the point after the blink peak where the signal reaches a similar amplitude as the initial velocity peak. |
| Closing Tent Duration | This corresponds to the time from blink start to the maximum peak velocity. This is from the start of the blink event to the point where the second derivative reaches its maximum. |
| Maximum Velocity to Peak Duration | This corresponds to the time between peak velocity and peak amplitude. This is the interval by measuring from the highest second derivative point (max velocity) to the first derivative zero crossing (peak amplitude). |
| Slope of Closing Tent | This corresponds to the slope of the closing phase up to maximum velocity. This is the change in amplitude over time from blink start to the point of maximum velocity. |
| Slope of Opening Tent | This corresponds to the slope of the opening phase after minimum velocity. This is the minimum velocity point to baseline, tracking the decrease in amplitude over time. |
| Slope at Closing Tent, Maximum Acceleration | This corresponds to the slope of the closing phase at the maximum acceleration of the closing tent, at the beginning of the signal’s peak phase. |
| Blink Phase Velocity Ratio | This corresponds to the ratio of velocities in closing and opening phases. This is calculated by dividing the closing velocity by the opening velocity. |
| Initial Blink Energy | This corresponds to the integral of the blink signal across the initial 5% of the blink, representing early energy. |
| Closing Phase Energy | This corresponds to the integral of the EOG signal over the eye-closing phase. Integrates the signal from the start of the blink to the peak. |
| Opening Phase Energy | This corresponds to the integral of the EOG signal over the eye-opening phase. Integrates from the peak to the return to baseline. |
| Closing Phase Slope Energy | This corresponds to the integral of the closing slope over time. Integrates the signal’s slope up to the peak velocity during closing. |
| Closing Phase Velocity Energy | This corresponds to the integral of the EOG signal from the left-side velocity peak up to the blink amplitude peak. It captures the energy or accumulated signal from the point where the closing velocity is at its maximum to the blink’s amplitude peak. |
| Opening Phase Velocity Energy | This corresponds to the integral of the EOG signal from the blink amplitude peak to the right-side velocity minimum. It represents the energy decay in the signal after the blink peak, spanning from the peak to where the opening velocity reaches its minimum. |
| Signal Average | This corresponds to the average value around the blink peak. |
| Acceleration Standard Deviation | This is the standard deviation of acceleration across the full blink. |
| Velocity Entropy | This corresponds to the entropy of blink velocity, indicating irregularities. |
| Acceleration Entropy | This corresponds to the entropy of blink acceleration, capturing the randomness in acceleration changes. |
| Maximum Acceleration Velocity Ratio | This is the ratio of maximum acceleration to maximum velocity in the opening phase of the peak. |
